# Towards early detection of adverse drug reactions: combining pre-clinical drug structures and post-market safety reports

**DOI:** 10.1186/s12911-019-0999-1

**Published:** 2019-12-18

**Authors:** Ruoqi Liu, Ping Zhang

**Affiliations:** 1 Department of Computer Science and Engineering, The Ohio State University, 2015 Neil Ave, Columbus, 43210 Ohio USA; 2 Department of Biomedical Informatics, The Ohio State University, 1800 Cannon Drive, Columbus, 43210 Ohio USA

**Keywords:** Adverse drug reactions, Signal Detection, FDA Adverse Event Reporting System, Drug similarity

## Abstract

**Background:**

Adverse drug reaction (ADR) is a major burden for patients and healthcare industry. Early and accurate detection of potential ADRs can help to improve drug safety and reduce financial costs. Post-market spontaneous reports of ADRs remain a cornerstone of pharmacovigilance and a series of drug safety signal detection methods play an important role in providing drug safety insights. However, existing methods require sufficient case reports to generate signals, limiting their usages for newly approved drugs with few (or even no) reports.

**Methods:**

In this study, we propose a label propagation framework to enhance drug safety signals by combining drug chemical structures with FDA Adverse Event Reporting System (FAERS). First, we compute original drug safety signals via common signal detection algorithms. Then, we construct a drug similarity network based on chemical structures. Finally, we generate enhanced drug safety signals by propagating original signals on the drug similarity network. Our proposed framework enriches post-market safety reports with pre-clinical drug similarity network, effectively alleviating issues of insufficient cases for newly approved drugs.

**Results:**

We apply the label propagation framework to four popular signal detection algorithms (PRR, ROR, MGPS, BCPNN) and find that our proposed framework generates more accurate drug safety signals than the corresponding baselines. In addition, our framework identifies potential ADRs for newly approved drugs, thus paving the way for early detection of ADRs.

**Conclusions:**

The proposed label propagation framework combines pre-clinical drug structures with post-market safety reports, generates enhanced drug safety signals, and can potentially help to accurately detect ADRs ahead of time.

**Availability:**

The source code for this paper is available at: https://github.com/ruoqi-liu/LP-SDA.

## Background

Adverse drug reactions (ADRs), identified as harmful and unintended reactions resulted from drug treatments, become main public health issues. Delayed detection of ADRs can cause a major damage to public health [1, 2] (e.g., accounting for significant amount of mortality and morbidity each year). It is estimated that over 2,000,000 serious ADRs occur among all hospitalized patients in the United States, which causes more than 100,000 deaths per year [2]. In addition, ADRs become the fourth leading cause of death in the United States, preceding serious medical events such as pulmonary disease, diabetes, AIDS and pneumonia [3]. Therefore, early detection of potential ADRs or drug safety signals can significantly reduce the health risk for patients and save money for additional hospital costs.

Though ADRs can be detected in both pre-marketing clinical trials and post-marketing surveillances, most ADR knowledges are revealed after the drugs being on market. Compared to clinical trials, post-marketing stage allows larger population and extended follow up. Real-world evidence, such as Spontaneous Reporting System (SRS) [4], Electronic Health Records (EHRs) [5], medical claims [6], social media and web search [7, 8], become important for detecting ADRs. Among those data sources, SRS remains a cornerstone of pharmacovigilance and are collected from a variety of sources, including healthcare providers, national authorities, pharmaceutical companies, medical literature and more recently directly from patients. SRS collects case reports such that each sample contains ADR status (Yes/No) and drug status (Yes/No). Such a structure allows SRS to be mined without an epidemiology design.

Due to the rich and valuable information offered by SRS data, a series of signal detection algorithms have been developed to detect drug safety signals from SRS. Proportional Reporting Rate (PRR) [9] and Reporting Odds Ratio (ROR) [10, 11] are the most commonly used methods, which are based on frequentist statistical analysis. And Multi-item Gamma Poisson Shrinker (MGPS) [12] and Bayesian Confidence Propagation Neural Network (BCPNN) [13]) are two Bayesian approaches that widely used for signal detection. Recently, another approach has emerged that combines pre-clinical drug structures with SRS to improve the original safety signals. Vilar et al. [14, 15] improve the original signals generated from health-care databases by incorporating biological and chemical information of drugs. Their methods firstly achieved improvement of performance in the analysis of two representative ADRs: rhabdomyolysis and pancreatitis. Vilar et al. [16] further demonstrate that other types of cheminformic similarity (e.g., 2D drug chemical structural similarity, adverse event profile similarity and target profile similarity) can also yield great results in the detection of drug safety signals. Moreover, Vilar et al. [17] present a 3D drug-ADR predictor, which incorporates 3D molecular structure similarity and drug-ADR standard reference, to improve ADRs identification and generate enriched drug-ADR signals. They apply the 3D drug-ADR predictor on SRS resources and find that the proposed predictor identifies more accurate signals than baseline methods. The underlying principle behind these approaches is that drugs with similar chemical structures are more likely to exhibit similar ADR [18]. In general, existing methods are developed to generate signals and/or re-rank original signals for drugs with enough reports in SRS, but few methods can be used to generate signals for newly approved drugs with few or even no safety reports in SRS.

There are some approaches that use machine learning techniques and pre-clinical information from large public drug databases to predict ADR [19–24]. Most of these methods typically use chemical, biological and phenotypic properties of drugs to build predictive models. In [19] for example, a computational approach is presented to predict the side effects of a given drug by incorporating information on other drugs and their side effects. They use drug-ADR pairs obtained from public drug databases both in the training process and performance evaluation. However, we just use these drug-ADR pairs as external evaluation resources which do not take part in the prior training process (A comparison of [19] and ours framework can be found in Fig. S1 of Additional file 1). To best of our knowledge, ours is the first signal detection framework that combines pre-clinical drug structures and post-market safety reports.

In this paper, we propose a label propagation framework to enhance drug safety signals by combining drug chemical structures with FDA Adverse Event Reporting System (FAERS) [25]. First of all, we compute original drug safety signals via common signal detection algorithms from FAERS. Then, we construct a drug-drug similarity network based on chemical structures. Finally, we generate enhanced drug safety signals by propagating original signals on the drug-drug similarity network. We apply the label propagation framework on four popular signal detection algorithms (PRR, ROR, MGPS, BCPNN) and find that our proposed framework can generate more accurate drug safety signals than the corresponding baseline methods. In addition, the proposed framework can identifies potential ADRs for newly approved drugs, thus providing promise for early detection of ADRs.

In general, the contributions of the paper lie in three-fold:

- We propose a label propagation framework to generate enhanced drug safety signals, which incorporates the pre-clinical drug structures with the post-market safety reports.
- We compare the proposed framework with four different state-of-the-art signal detection algorithms and evaluate the performance in detecting ADRs.
- We also apply our framework on newly approved drugs (with few cases in SRS) and access whether pre-clinical drug structures can help to early detect safety signals prior to FDA safety label change.

## Methods

### Datasets

#### FAERS database

**Fig. 1:**
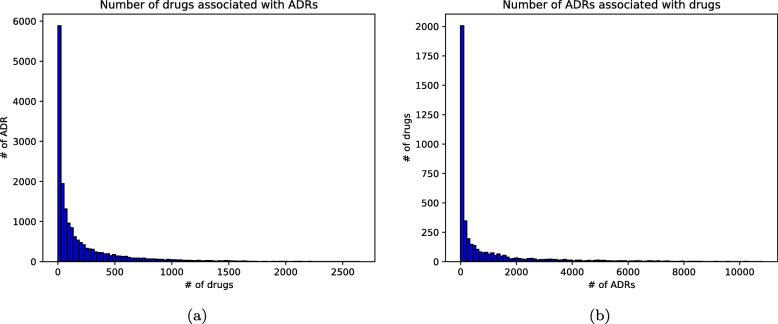
The frequencies of ADRs and drugs. **a** Frequencies of number of drugs associated with each ADR, **b** Frequencies of number of ADRs associated with each drug

The SRS data used in this work is FAERS. we adopt a curated and standardized version of FAERS data from 2004 to 2014 [26]. After removing duplicate case records, mapping drug names to RxNorm concepts and ADR outcomes to Medical Dictionary for Regulatory Activities (MedDRA) codes [27], we obtain 4245 unique drugs, 17,671 ADRs and totalling 4,928,413 reports. We plot the frequencies of ADRs and drugs of FAERS data in Fig. 1 to demonstrate the data distribution of this dataset. The number of drugs associated with ADRs varies a lot with an average of 213 as shown in Fig. 1a. And the number of ADRs associated with each drug with an average of 887 in Fig. 1b.

#### Pubchem database

PubChem Compound database [28] provides unique chemical structure information of drugs. We map the concept IDs of drugs in FAERS into PubChem IDs using the exact drug names and then extract the drug chemical substructures from PubChem. Among 4245 unique drugs in FAERS, 2708 drugs are mapped and their chemical features are extracted from PubChem.

#### SIDER ground truth data

The Side Effect Resource (SIDER) database [29] contains approved drugs and their recorded ADRs, which are collected from package inserts (i.e., drug labels). In the SIDER version 4.1, it contains totalling 1430 drugs, 5868 ADRs and 139,756 drug-ADR pairs. We use drug-ADR pairs extracted from SIDER version 4.1 as positive controls for evaluation. Of 2708 drugs with chemical features, 843 drugs are mapped to SIDER by converting PubChem IDs to STITCH IDs in SIDER. ADRs in SIDER are recorded in both Lowest Level Terms (LLT) and Preferred Terms (PT) form of MedDRA. We select PT for ADRs as our evaluation dataset. Thus, we end up with 843 drugs, 842 ADRs and 65,636 drug-ADR pairs as the ground truth data in the experiment.As further validation of the approach, we also use OFFSIDES [30], a post-marketing dataset to test the performance (See Table S4 in Additional file 1).

### Overall framework

**Fig. 2:**
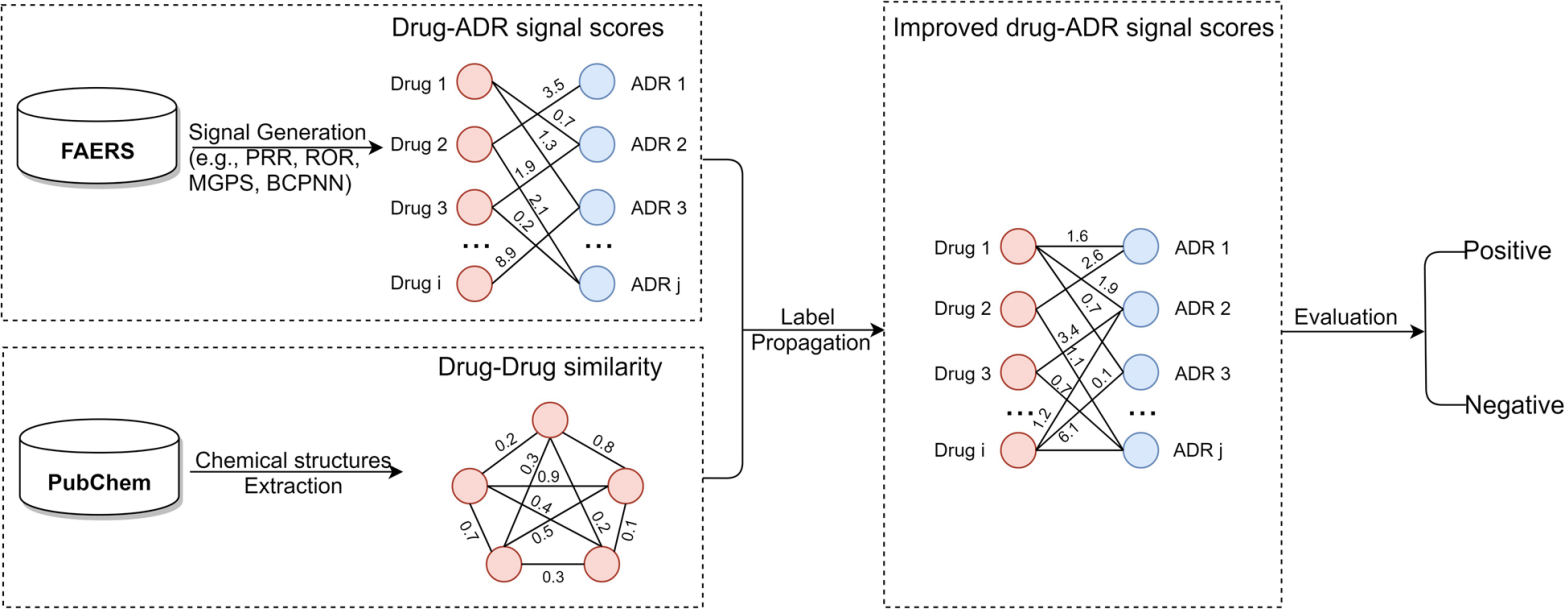
The overall framework. It consists of three main steps: computing original drug safety signals, constructing a drug-drug similarity network and generating enhanced drug safety signals through a label propagation process

The overall framework of this paper is outlined in Fig. 2. It consists of three main steps: computing original drug safety signals from FAERS reports, constructing a drug-drug similarity network from pre-clinical drug structures, and generating enhanced drug safety signals through a label propagation process.

### Computing drug safety signals

Our study covers four commonly used signal detection algorithms. Table 1 lists the main properties of each algorithm. The proportional reporting ration (PRR) [9] and the reporting odds ratio (ROR) [10, 11] are two popular measurements of frequentist statistical methods. For each drug-adverse pair, we construct a 2 ×2 contingency table (Table 2) and compute the signal scores as follow:

1 $$ PRR = \frac{a/a+c}{b/b+d}  $$

**Table 1:** Common disproportionality analysis for safety signals

	Methods	Description	Signal score computation
Frequentist statistical methods	Proportional Reporting Ratio (PRR)	Statistical method to calculate the relative risk in order to measure the association strength for a drug-ADR pair	PRR05: lower bound of the 95% confidence interval of relative risk reporting ratio distribution
	Reporting Odds Ratio (ROR)	Statistical method to calculate the odds ratio in order to measure the association strength for a drug-ADR pair	ROR05: lower bound of the 95% confidence interval of odds ratio distribution
Bayesian-based methods	Multi-item Gamma Poisson Shrinker (MGPS)	Bayesian-based method to prevent false-positive signals from multiple comparisons. Generate an adjusted value based on Reporting Ratio (RR)	EB05: lower bound of the 95% of the posterior distribution for RR
	Bayesian Confidence Propagation Neural Network (BCPNN)	Bayesian-based method to prevent false-positive signals from multiple comparisons. Generate an adjusted value based on Information Component (IC)	BCPNN25: lower bound of the 2.5% of the posterior distribution for IC

**Table 2:** 2 ×2 contingency table for a drug-ADR pair

	Reports with ADR	Reports without ADR	Total
Reports with drug	*a*	*b*	*a*+*b*
Reports without drug	*c*	*d*	*c*+*d*
Total	*a*+*c*	*b*+*d*	*a* + *b* + *c* + *d*

2 $$ ROR = \frac{a/c}{b/d}  $$

In this paper, we use PRR05 (referred as PPR) and ROR05 (referred as ROR) as baseline methods in the experiments. The multi-item gamma poisson shrinker (MGPS) [12, 31] and bayesian confidence propagation neural network (BCPNN) [13] are widely used Bayesian approaches for signal detection. We adopt EB05 of MGPS and BCPNN25 of BCPNN as our baseline methods.

### Constructing drug similarity network

We construct a drug similarity network based on chemical structures. To be specific, we treat different drugs as nodes on the network, and compute edge weights on the network with drug chemical structure similarities. The similarity is based on a chemical structure fingerprint corresponding to the 881 chemical substructure [32] defined in PubChem. Each drug can be represented by an 881-dimensional binary profile whose elements indicate the presence or absence of corresponding PubChem substructures with value 1 or 0. The Jaccard similarity between two drugs can be calculated by:

3 $$ Jaccard(A, B) = \frac{|A\cap B|}{|A \cup B|}  $$

where A and B denote the profiles of two drugs.

### Generating enhanced drug safety signals

Label propagation algorithms are widely adopted in analyzing weighted *N* nodes graph to discover latent information [33] and have been applied to biomedical problems [34]. At the beginning of the algorithms, a small portion of nodes have labels and these labels are propagated to previously unlabeled nodes through the algorithms.

In our method, we generate enhanced drug safety signals via propagating original signals on the drug similarity network. The weighted *N* nodes graph is constructed based on the *N*×*N* drug similarity matrix *A*, where *A*_*i*,*j*_≥0 represents the similarity for drug *i* and drug *j*. Drugs are treated as nodes in the graph and the edge weights are assigned by the drug similarities. The signal score matrix *S* of drug-ADR pairs, where *S*_*i*,*j*_ denotes the signal score of drug*i*-ADR*j* combination, are considered as initial labels of nodes. For the drug *D*_*i*_, the initial labels are *i*th row of the signal scores matrix *S*, which are denoted as *S*_*i*_. The label information of initial drug nodes is propagated to the nodes through the weighted edges in the graph by an iterative approach. To guarantee the convergence of the updates, the original drug similarity matrix *A* needs to be normalized so that the row sum is one. We denote the normalized matrix as *W*.

Using *W*, we propagate labels from the labeled drug nodes to the unlabeled nodes. In every iteration, the label information of each node is updated by absorbing labels from its neighbors by a probability *γ*, and retaining labels of its previous labels by a probability (1−*γ*). The updating formula for a drug node *i* in the *t* th iteration from step *t*−1 to step *t* can be denoted as below,

4 $$ Y_{i}^{t} = \gamma WY_{i}^{t-1} + (1-\gamma)S_{i}  $$

In this formula, $Y_{i}^{t}$ represents the updated label information of drug node *i* in *t*th iteration, and 0<*γ*<1 is the absorbing probability that determine the label information absorbed from neighbors. By considering all drug nodes at the same time, we can formulate the updating formula (4) into a matrix form,

5 $$ Y^{t} = \gamma WY^{t-1} + (1-\gamma)S  $$

After *t* iterations, (5) can be written as,

6 $$ Y^{t} = (\gamma W)^{t}S + (1-\gamma)\sum_{i=0}^{t-1}(\gamma W)^{i}S  $$

Since $\sum _{j=0}^{N}A_{i,j}=1$, the spectral radius *ρ*(*W*)≤1. And 0<*γ*<1, thus ${\lim }_{t\to \infty }(\gamma W)^{t}=0$ and ${\lim }_{t\to \infty }\sum _{i=0}^{t-1}(\gamma W)^{i}=(I-\gamma W)^{-1}$, where *I* is the identity matrix of order *N*. Therefore, the iteration of updating formula will converge as (The proof of convergence can be found in [33]),

7 $$ Y={\lim}_{t\to\infty}Y^{t}=(1-\gamma)(I-\gamma W)^{-1}S  $$

where *Y* is the final label information for *N* drug nodes and *S* is the matrix for initial label information.

To generate signals for a new drug, we regard the signals of the drug with all ADRs as 0. Then we calculate the similarities between new drugs and other drugs. Based on current similarity network, we can generate safety signals via label propagation, even there is no existing report.

In general, the original signal scores computed by common signal detection algorithms are further improved through the label propagation on the drug similarity network. The final labels (scores) can be regarded as the improved signals for drug-ADR pairs.

## Results

### Experiment setup

The known drug-ADR pairs extracted from SIDER are treated as positive controls, and the unknown drug-ADR pairs are referred as negative controls. Since the number of positive samples is much fewer than negative ones, we randomly sample part of negative controls from all unknown pairs. The size of negative samples is twice the size of positive controls. To fully demonstrate the performance of our methods, we also compile an evaluation dataset with all drug-ADR pairs from SIDER as reference positives and the complement set of SIDER drug-ADR pairs as reference negatives (i.e., without any sub-sampling of negatives). We conduct the experiments on this alternative dataset and report the results in Table S2 of Additional file 1.

In the performance comparison, we use Area Under the Curve (AUC) score, Area Under the Precision-Recall Curve (AUPR) score, precision, recall, accuracy and F1-score (F1) for performance comparison. AUC score is a graphical figure of true positive rate (TPR) and false positive rate (FPR), which can be plotted by varying the threshold value for output scores. The definition of TPR and FPR shows below:

8 $$ \left\{ \begin{array}{lr} \text{TPR}=\frac{\text{True Positive}}{\text{True Positive}+\text{False Negative}} & \vspace{1ex} \\ \text{FPR}=\frac{\text{False Positive}}{\text{False Positive}+\text{True Negative}} & \end{array} \right.  $$

Similarity, AUPR can be plotted in the same way based on precision and recall score. Precision measures the probability of the output identified safety signals being correct. Recall measures the probability of real true safety signals being estimated as the outputs. The equations of precision and recall are shown in 9.

9 $$  \left\{ \begin{array}{lr} \text{Precision}=\frac{\text{True Positive}}{\text{True Positive}+\text{False Positive}} & \vspace{1ex} \\ \text{Recall}=\frac{\text{True Positive}}{\text{True Positive}+\text{False Negative}} & \end{array} \right.  $$

Accuracy measures the probability of all ground labels of drug-pairs being estimated correctly. F1 is defined as the harmonic mean of precision and recall:

10 $$ \text{F1} = \frac{2*\text{Precision}*\text{Recall}}{\text{Precision}+\text{Recall}}  $$

There is one parameter: absorbing probability (*γ*) of label propagation in the proposed method. We consider *γ* in {0.1,0.2,0.3,...,0.9} and build the model with *γ* that yields the maximum AUC score. We evaluate the performance of models on different parameters and show the results in the Fig. S2 of Additional file 1. The optimal values of *γ* for each signal detection algorithms are shown in Table S3 of Supplementary Materials.

### Performance evaluation on all ADRs

We compare the proposed methods with four baselines (PRR, ROR, MGPS, BCPNN) using all years data and report the six metrics in Table 3. “LP-Method name” denotes the proposed method and which signal detection algorithm we use to generate original signals. From Table 3, we can observe that among these four signal detection algorithms, MGPS outperforms other baseline methods resulting in the best AUC scores and AUPR scores. And our methods are better than all the corresponding baseline methods in terms of AUC scores, AUPR scores and precision. The results demonstrate that drug-drug similarities can help to enhance the safety signals since the similar drugs may induce same ADRs. By this way, the original drug safety signals are improved by incorporating information from similar drugs.

**Table 3:** Comparison of the proposed methods and corresponding baseline methods on all years reports

Method	AUC	AUPR	Precision	Recall	Accuracy	F1
PRR	0.716	0.517	0.786	0.466	0.629	0.586
**LP-PRR**	**0.728**	**0.534**	**0.801**	**0.478**	**0.644**	**0.588**
ROR	0.716	0.518	0.786	0.466	0.629	0.585
**LP-ROR**	**0.728**	**0.534**	**0.801**	**0.477**	**0.643**	**0.588**
MGPS	0.727	0.544	0.746	0.483	0.649	0.586
**LP-MGPS**	**0.751**	**0.574**	**0.770**	**0.498**	**0.665**	**0.601**
BCPNN	0.670	0.445	0.867	0.428	0.570	0.573
**LP-BCPNN**	**0.671**	**0.449**	**0.911**	**0.428**	**0.574**	**0.573**

Evaluation metrics of fixed levels of sensitivities and specificities values can be found in Table S1 of Additional file 1. The bold in the table is maximum values of that evaluation metrics on different methods

**Fig. 3:**
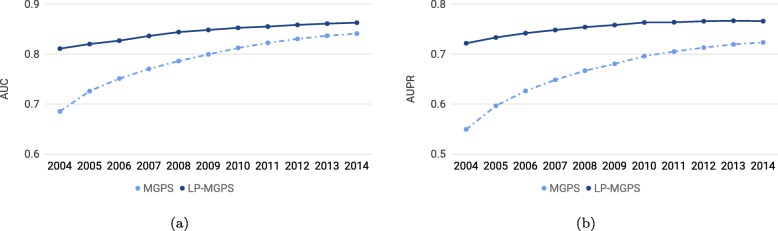
Comparison of the proposed method with MGPS on yearly cumulative reports. **a**: AUC scores of LP-MGPS and MGPS with yearly reports, **b**: AUPR scores of LP-MGPS and MGPS with yearly reports

We also plot the yearly change curve for LP-MGPS and MGPS based on AUC scores and AUPR scores in Fig. 3. Here, 2004,2005,...,2014 of horizontal axis represent the reports we use to generate signals accumulated from 2004 to current year (i.e., 2008 denotes reports from 2004 to 2008 are utilized to generate signals). According to Fig. 3, we can find that our method LP-MGPS outperforms its corresponding baseline MGPS on every cumulative years. In addition, the proposed method can achieve better performance especially only with reports of early years.

### Performance evaluation on representative ADRs

To further characterize the performance of the proposed method, we select ADRs from Designated Medical Event (DME) [35] for additional comparisons. DME contains standardized medical concept terms released by The European Medicines Agency (EMA), which is a list of inherently serious ADRs. We map the ADRs of DME with our datasets and remove the ADRs associated with less than 10 drugs. 31 ADRs are considered for performance evaluation and Table 4 shows the comparison of proposed LP-MGPS and the original MGPS algorithm on top 15 ADRs ranked by AUPR scores. “Number of positive drugs” denotes the number of drugs that associated with each ADR. Here, we use MGPS as our based signal detection algorithm since it yields highest AUC and AUPR scores for this task. According to the results, the proposed method is better than the corresponding baseline method on all 15 ADRs in terms of AUPR scores. And our methods outperform the baseline on most cases for AUC scores. (More experiments on these representative ADRs can be found in Table S5 and Table S6 of Additional file 1).

**Table 4:** Top 15 ADRs ranked by AUPR

ADR concept ID	ADR name	Number of positive drugs	AUPR		AUC	
			MGPS	LP-MGPS	MGPS	LP-MGPS
36009756	Anaphylactic reaction	373	0.968	**0.973**	0.779	**0.798**
35104877	Febrile neutropenia	52	0.968	**0.972**	0.955	**0.962**
35707713	Pancreatitis	197	0.956	**0.959**	0.862	**0.865**
36009762	Angioedema	328	0.949	**0.955**	0.794	**0.807**
35406359	Deafness	123	0.932	**0.940**	0.819	**0.832**
37019318	Renal failure	207	0.937	**0.939**	0.824	**0.828**
36009760	Anaphylactoid shock	151	0.869	**0.928**	0.681	**0.756**
35104879	Granulocytopenia	224	0.901	**0.925**	0.756	**0.789**
36009724	Stevens-Johnson syndrome	209	0.917	**0.922**	0.815	**0.825**
36516888	Rhabdomyolysis	90	0.914	**0.920**	0.866	**0.868**
35104103	Bone marrow failure	195	0.914	**0.920**	**0.758**	0.756
36009707	Erythema multiforme	252	0.911	**0.918**	0.777	**0.782**
35104281	Haemolytic anaemia	128	0.901	**0.916**	**0.788**	0.785
35909518	Hepatic failure	136	0.910	**0.915**	0.813	**0.820**
35104101	Aplastic anaemia	109	0.885	**0.913**	0.748	**0.802**

The bold in the table is maximum values of that evaluation metrics

## Discussion

A label propagation framework is built in this study, which enriches post-market safety reports with pre-clinical drug similarity network to generate enhanced safety signals. The overall performance of the proposed method is superior, the performance on those important ADRs are good, and the MGPS-based method achieves the best performance.

**Fig. 4:**
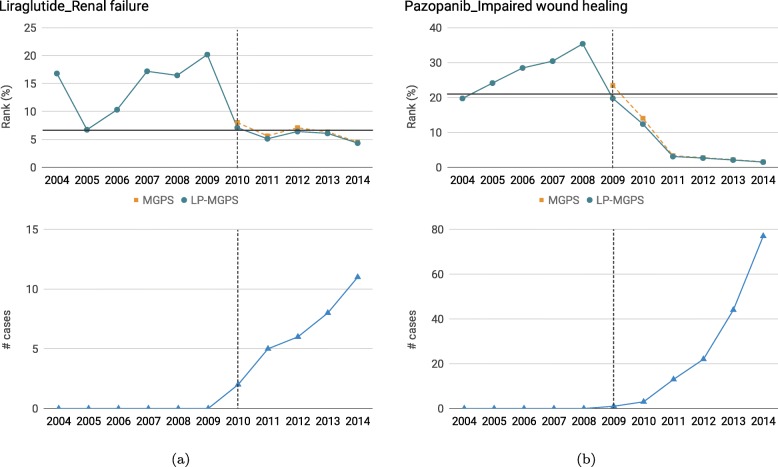
Comparison of the proposed method with MGPS on newly approved drugs: **a** yearly rankings change of Liraglutide-Renal failure, and the label change happens in 2011, **b** yearly rankings change of Pazopanib-Impaired wound healing, and the label change happens in 2014

We further demonstrate the performance of the proposed method on newly approved drugs which have few (or even no) reports in SRS. The safety related labels for a drug are released by FDA since the drug approval and ADRs are recorded in labeling information for drugs. The labeling information might be revised quarterly by port-marketing surveillance. Here, we report the performance of ADRs detection for two recently approved drugs “liraglutide” and “pazopanib” in Fig. 4. We use MGPS-based method to generate original signals since we obtain the best performance on MGPS. We compute the yearly rankings of the drug to the ADR and the number of drug-ADR cases in SRS. The horizontal axis here represents the cumulative years from 2004 to current year. The rank in vertical axis denotes the percentile of the drug ranking, which can be calculated by $\frac {\text {rank of the drug}}{\text {\# all drugs}} * 100$ after sorting the entire drug list in a descending order.

Liraglutide is a medication used to treat diabetes or obesity [36], and it is approved for medical use in the United States in 2010 [37] and in Europe in 2009 [38]. In 2011, renal failure was updated to the labeling information of liraglutide [39]. According to Fig. 4a, we can find that Liraglutide-Renal failure first showed up in SRS in 2010 and accumulated to 11 cases in 2014. Thus, the baseline which entirely rely on the sufficient cases can only generate signals for this pair after 2010. The ranking of liraglutide gradually increases as more years data accumulated. The proposed method performs better than the baseline after 2010. More importantly, the proposed method is able to generate signals before 2010 and can predict liraglutide to cause renal failure as early as of 2005 by taking the case reports of liraglutide’s similar drugs into the consideration. Therefore, the proposed method can early detect the safety-related labeling changes than the labels revised by FDA.

Pazopanib is a medicine used for treatment of advanced renal cell carcinoma (RCC) and advanced soft tissue sarcoma (STS) [40]. It is approved for medical use in the United States in 2009 [41] and in Europe in 2010 [42]. The impaired wound healing was included in one of syndromes in labeling information of pazopanib in 2014 [43]. For Pazopanib-Impaired wound healing shown in Fig. 4b, it is initially reported by SRS in 2009 and continually accumulated up to 77 cases by 2014. The baseline can not generate signals for Pazopanib-Impaired wound healing without any cases. However, the proposed method is able to identify potential safety signals before 2009 and yearly rankings of the pazopanib confirm that our method can detect the safety signals prior to FDA safety label change.

The above instances confirm that the algorithm is able to detect drug safety signal before the approval, and consistently outperforms the state-of-the-art in early detection and before the drug label change which every pharmacy is trying to avoid.

## Conclusions

In this paper, we present a label propagation framework, which integrates drug chemical information with post-market safety reports, to generate enhanced drug safety signals. The drug safety signals are enhanced through the process of label propagation with the drug similarity computed from the chemical information. We compare the performance of our methods with four different state-of-the-art signal detection algorithms (PRR, ROR, MGPS, BCPNN) using safety reports from SRS. The results demonstrate that the proposed methods outperform their corresponding baselines in generating accurate drug safety signals. Extensive experiments show that our methods are able to accurately detect potential ADRs for newly approved drugs with few safety reports, which pave the way for early detection of ADRs.

This study can be extended in multiple directions in the future in terms of both drug features and post-market real-world evidence. Other types of available data sources of drugs such as chemical-protein binding and therapeutic indication data can be leveraged for the construction of drug similarity networks. Furthermore, the label propagation framework can be applied to enhance drug safety signals generated by other real-world evidence such as EHRs and medical claims.

## Supplementary information

**Additional file 1** Additional experimental results. Figure S1. Comparison of our framework and existing side effect prediction framework. Figure S2 AUC scores of proposed methods using different parameter values. TableS1-S3. Additional results for performance evaluation on all ADRs. Table S4. Performance Evaluation on All ADRs using OFFSIDE as Ground Truth. Table S5-S6 Performance Evaluation on representative ADRs

## Data Availability

The datasets used and analyzed during the current study are available from the curated FAERS [26], SIDER [29]. The code is available at https://github.com/ruoqi-liu/LP-SDA. All datasets and software used in this study are fully accessible, free of charge.

## Abbreviations

ADRs: Adverse drug reactions
FAERS: FDA’s adverse event reporting system
PRR: Proportional Reporting Ratio
ROR: Reporting Odds Ratio
MGPS: Multi-item Gamma Poisson Shrinker
BCPNN: Bayesian Confidence Propagation Neural Network
