# Supplementary material for: Towards early detection of adverse drug reactions: combining pre-clinical drug structures and post-market safety reports
Source: BMC Med Inform Decis Mak. 2019 Dec 18;19:279. doi: 10.1186/s12911-019-0999-1 (PMC6918608; doi:10.1186/s12911-019-0999-1)
Supplement: Supplementary file 1 — Additional file 1 Additional experimental results. Figure S1. Comparison of our framework and existing side effect prediction framework. Figure S2 AUC scores of proposed methods using different parameter values. TableS1-S3. Additional results for performance evaluation on all ADRs. Table S4. Performance Evaluation on All ADRs using OFFSIDE as Ground Truth. Table S5-S6 Performance Evaluation on representative ADRs [file 12911_2019_999_MOESM1_ESM.docx]

**A Framework Comparison**

Figure S1 shows the difference between ours signal detection framework and side effect prediction framework in Atias et al [1]. As shown in Figure 1, we use drug-ADR pairs extracted from SIDER as external evaluation dataset while Atias et al [1]. use SIDER to generate drug-ADR association matrix, ADR similarity matrix and initial labels for label propagation.


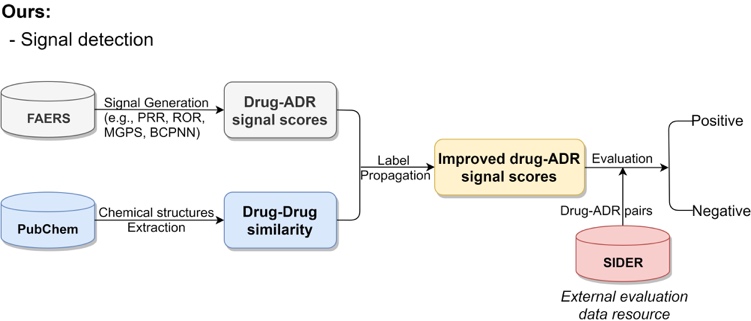

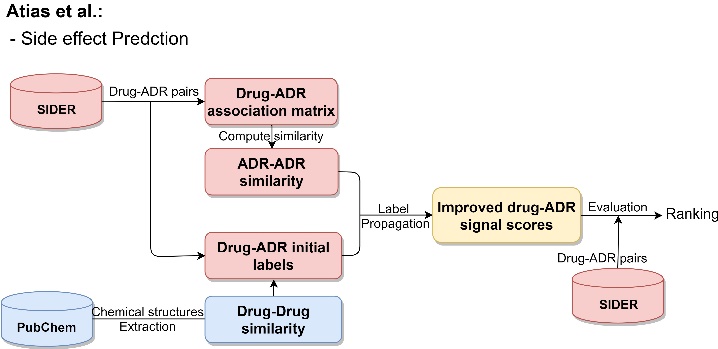


**Figure S1** Comparison of our framework and side effect prediction framework in [3]

**B Additional Results for Performance Evaluation on all ADRs**

To extend our evaluation metrics, we also use fixed levels of sensitivity and specificity to test the performance on all ADRs of all year’s reports in Table S1. According to Table S1, our proposed label propagation based methods perform better than the corresponding baselines. Different baseline algorithms yield different performance on ADR detection. Among all four algorithms, MGPS generate the most accurate signal scores with highest specificity values under fixed levels of sensitivity (highest sensitivity values under fixed levels of specificity as well).

**Table S1** Comparison of proposed method and corresponding baselines on all years reports based on fixed levels of sensitivity and specificity values

|  | Specificity | | | | | | | |
| --- | --- | --- | --- | --- | --- | --- | --- | --- |
| Sensitivity | PRR | LP-PRR | ROR | LP-ROR | MGPS | LP-MGPS | BCPNN | LP-BCPNN |
| 0.5 | 0.784 | 0.800 | 0.783 | 0.799 | 0.800 | 0.827 | 0.704 | 0.703 |
| 0.6 | 0.718 | 0.734 | 0.718 | 0.733 | 0.732 | 0.761 | 0.642 | 0.642 |
| 0.7 | 0.639 | 0.650 | 0.639 | 0.649 | 0.648 | 0.678 | 0.574 | 0.574 |
| 0.8 | 0.534 | 0.531 | 0.533 | 0.531 | 0.526 | 0.562 | 0.492 | 0.491 |
| 0.9 | 0.371 | 0.372 | 0.371 | 0.372 | 0.371 | 0.391 | 0.371 | 0.360 |
|  | Sensitivity | | | | | | | |
| Specificity | PRR | LP-PRR | ROR | LP-ROR | MGPS | LP-MGPS | BCPNN | LP-BCPNN |
| 0.5 | 0.825 | 0.821 | 0.825 | 0.821 | 0.819 | 0.842 | 0.792 | 0.790 |
| 0.6 | 0.741 | 0.747 | 0.741 | 0.747 | 0.745 | 0.769 | 0.663 | 0.661 |
| 0.7 | 0.624 | 0.644 | 0.624 | 0.643 | 0.641 | 0.676 | 0.506 | 0.505 |
| 0.8 | 0.471 | 0.500 | 0.471 | 0.499 | 0.500 | 0.545 | 0.333 | 0.332 |
| 0.9 | 0.267 | 0.285 | 0.267 | 0.285 | 0.304 | 0.349 | 0.173 | 0.174 |

We also compile an alternative evaluation dataset for performance evaluation. We consider all known drug-ADR pairs in SIDER as positive labels and all unknown pairs of drug-ADR in SIDER as our negative labels. There are 1430 drugs, 5868 ADRs and 139756 drug-ADR pairs in SIDER. Thus, the ratio of positives and negatives is $139756 / (1430*5868) =0.0166 \approx1:100$. We randomly sample 50% of the entire evaluation dataset as validation set and 50% as testing set. The validation set is used for tuning model parameter $\gamma$ of label propagation. We then evaluate the performance on the testing set using the optimal value of parameter $\gamma$ selected by validation process. We conduct experiments on all years reports and show the evaluation results on both validation set and testing set in Table S2. We can observe that the proposed methods outperform the corresponding baselines in most evaluation metrics. Table S3 shows the optimal value of parameter $\gamma$ used for performance evaluation on testing set.

**Table S2** Comparison of proposed method and corresponding baselines on all years reports with validation/test split.

|  | AUC | | AUPR | | Precision | | Recall | | Accuracy | | F1 | |
| --- | --- | --- | --- | --- | --- | --- | --- | --- | --- | --- | --- | --- |
| Method | Validation | Test | Validation | Test | Validation | Test | Validation | Test | Validation | Test | Validation | Test |
| PRR | 0.717 | 0.719 | 0.186 | 0.184 | 0.554 | 0.560 | 0.184 | 0.183 | 0.732 | 0.728 | 0.277 | 0.276 |
| **LP-PRR** | 0.729 | 0.730 | 0.196 | 0.193 | 0.522 | 0.512 | 0.200 | 0.202 | 0.762 | 0.768 | 0.289 | 0.290 |
| ROR | 0.717 | 0.718 | 0.186 | 0.186 | 0.556 | 0.505 | 0.183 | 0.196 | 0.729 | 0.763 | 0.275 | 0.282 |
| **LP-ROR** | 0.729 | 0.730 | 0.195 | 0.196 | 0.525 | 0.555 | 0.198 | 0.200 | 0.759 | 0.753 | 0.289 | 0.294 |
| MGPS | 0.730 | 0.709 | 0.206 | 0.196 | 0.519 | 0.436 | 0.203 | 0.203 | 0.767 | 0.789 | 0.292 | 0.277 |
| **LP-MGPS** | 0.752 | 0.740 | 0.225 | 0.216 | 0.476 | 0.464 | 0.237 | 0.226 | 0.810 | 0.803 | 0.317 | 0.304 |
| BCPNN | 0.669 | 0.675 | 0.141 | 0.143 | 0.720 | 0.737 | 0.142 | 0.146 | 0.572 | 0.576 | 0.237 | 0.243 |
| **LP-BCPNN** | 0.670 | 0.676 | 0.142 | 0.144 | 0.697 | 0.732 | 0.143 | 0.146 | 0.585 | 0.578 | 0.237 | 0.243 |

**Table S3** Optimal parameter $\gamma$ used for performance evaluation on testing set

| Method | $\gamma$ |
| --- | --- |
| LP-PRR | 0.5 |
| LP-ROR | 0.5 |
| LP-MGPS | 0.7 |
| LP-BCPNN | 0.1 |

**C Performance Evaluation on All ADRs using OFFSIDE as Ground Truth**

As further validation of our methods, we use OFFSIDE dataset to test whether our methods can accurately detect drug safety signals. We conduct the same experiments with validation/test split on all years’ reports. In Table S4, we show the evaluation results based on six metrics on validation and test sets. We find that the proposed method performs better than the corresponding baseline method as well on OFFSIDE dataset.

**Table S4** Comparison of proposed method and corresponding baselines on all years reports with validation/test split.

|  | AUC | | AUPR | | Precision | | Recall | | Accuracy | | F1 | |
| --- | --- | --- | --- | --- | --- | --- | --- | --- | --- | --- | --- | --- |
| Method | Validation | Test | Validation | Test | Validation | Test | Validation | Test | Validation | Test | Validation | Test |
| PRR | 0.792 | 0.794 | 0.468 | 0.477 | 0.646 | 0.627 | 0.421 | 0.436 | 0.765 | 0.776 | 0.510 | 0.514 |
| **LP-PRR** | 0.793 | 0.800 | 0.470 | 0.482 | 0.658 | 0.635 | 0.416 | 0.432 | 0.760 | 0.773 | 0.509 | 0.514 |
| ROR | 0.793 | 0.789 | 0.471 | 0.468 | 0.640 | 0.641 | 0.423 | 0.429 | 0.767 | 0.771 | 0.510 | 0.514 |
| **LP-ROR** | 0.796 | 0.788 | 0.473 | 0.471 | 0.648 | 0.629 | 0.420 | 0.434 | 0.764 | 0.775 | 0.509 | 0.514 |
| MGPS | 0.794 | 0.799 | 0.484 | 0.487 | 0.639 | 0.665 | 0.427 | 0.413 | 0.769 | 0.758 | 0.512 | 0.510 |
| **LP-MGPS** | 0.795 | 0.801 | 0.487 | 0.489 | 0.646 | 0.652 | 0.422 | 0.417 | 0.766 | 0.762 | 0.511 | 0.509 |
| BCPNN | 0.762 | 0.766 | 0.391 | 0.424 | 0.720 | 0.718 | 0.354 | 0.370 | 0.699 | 0.715 | 0.475 | 0.488 |
| **LP-BCPNN** | 0.763 | 0.779 | 0.393 | 0.424 | 0.725 | 0.707 | 0.352 | 0.370 | 0.695 | 0.718 | 0.474 | 0.493 |

**D Parameters**

We consider the absorbing probability ($\gamma$) of label propagation in range $\{0.1, 0.2,\ldots0.9\}$ and plot the AUC scores under different parameters based on all years reports in Figure S2. As observed, parameters have consistent influences on AUC scores of four signal detection algorithms. Performances of these methods increase as the $\gamma$ increases, and then decrease after reaching a peak (approximately $\gamma$=0.6 for PRR, ROR, GPS and $\gamma$=0.2 for BCPNN). For every ending year reports (Figure 3 in paper), we use the parameters that reach the highest AUC scores. And in performance evaluation on representative ADRs, we use LP-MGPS and apply $\gamma$=0.6 for generating results.


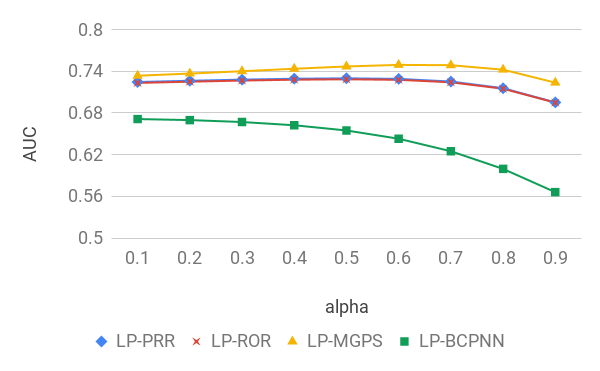


**Figure S2** AUC scores of proposed methods using different parameter values

**E Performance Evaluation on All 31 Representative ADRs**

**Table S5** Comparison of proposed method and corresponding baselines on all years reports with validation/test split.

| ADR ID | ADR Name | # positive drugs | AUPR (MGPS) | AUPR (LP-MGPS) | AUC (MGPS) | AUC (LP-MGPS) |
| --- | --- | --- | --- | --- | --- | --- |
| 35909513 | Acute hepatic failure | 14 | 0.670 | **0.677** | **0.891** | 0.890 |
| 36009756 | Anaphylactic reaction | 373 | 0.968 | **0.973** | 0.779 | **0.798** |
| 36009760 | Anaphylactoid shock | 151 | 0.869 | **0.928** | 0.681 | **0.756** |
| 36009762 | Angioedema | 328 | 0.949 | **0.955** | 0.794 | **0.807** |
| 35104100 | Aplasia pure red cell | 13 | **0.701** | 0.691 | 0.868 | **0.893** |
| 35104101 | Aplastic anaemia | 109 | 0.885 | **0.913** | 0.748 | **0.802** |
| 37019322 | Azotaemia | 39 | 0.679 | **0.688** | **0.769** | 0.745 |
| 35607441 | Blindness | 38 | 0.861 | **0.866** | 0.886 | **0.889** |
| 35104103 | Bone marrow failure | 195 | 0.914 | **0.920** | **0.758** | 0.756 |
| 35406359 | Deafness | 123 | 0.932 | **0.940** | 0.819 | **0.832** |
| 37320166 | Dermatitis exfoliative generalised | 143 | 0.789 | **0.818** | **0.561** | 0.543 |
| 36009707 | Erythema multiforme | 252 | 0.911 | **0.918** | 0.777 | **0.782** |
| 35104877 | Febrile neutropenia | 52 | 0.968 | **0.972** | 0.955 | **0.962** |
| 35104879 | Granulocytopenia | 224 | 0.901 | **0.925** | 0.756 | **0.789** |
| 35104302 | Haemolysis | 11 | 0.774 | **0.789** | 0.917 | **0.923** |
| 35104281 | Haemolytic anaemia | 128 | 0.901 | **0.916** | **0.788** | 0.785 |
| 35909518 | Hepatic failure | 136 | 0.910 | **0.915** | 0.813 | **0.820** |
| 35909611 | Hepatic necrosis | 57 | 0.769 | **0.796** | 0.753 | **0.779** |
| 35909618 | Hepatitis fulminant | 17 | 0.570 | **0.592** | 0.832 | **0.859** |
| 35708302 | Intestinal perforation | 27 | 0.688 | **0.700** | 0.789 | **0.793** |
| 35707713 | Pancreatitis | 197 | 0.956 | **0.959** | 0.862 | **0.865** |
| 35707714 | Pancreatitis acute | 41 | 0.808 | **0.809** | 0.845 | **0.846** |
| 37219844 | Pulmonary fibrosis | 37 | 0.817 | **0.827** | 0.870 | **0.879** |
| 37219868 | Pulmonary hypertension | 28 | **0.677** | 0.675 | **0.815** | 0.813 |
| 37019318 | Renal failure | 207 | 0.937 | **0.939** | 0.824 | **0.828** |
| 36516888 | Rhabdomyolysis | 90 | 0.914 | **0.920** | 0.866 | **0.868** |
| 36009724 | Stevens-Johnson syndrome | 209 | 0.917 | **0.922** | 0.815 | **0.825** |
| 35104694 | Thrombotic thrombocytopenic purpura | 20 | 0.671 | **0.671** | **0.888** | 0.871 |
| 35204977 | Torsade de pointes | 61 | 0.887 | **0.903** | 0.855 | **0.870** |
| 36009754 | Toxic epidermal necrolysis | 175 | 0.878 | **0.885** | 0.735 | **0.748** |
| 35204981 | Ventricular fibrillation | 72 | 0.809 | **0.826** | 0.813 | **0.834** |

**F Performance Evaluation on Representative ADRs using all six metrics**

We conduct experiments on representative ADRs with all six metrics. In this case, we consider all negatives without any sub-sampling with all positives as our evaluation dataset. Table S5 shows the results of Top 15 ADRs ranked by AUC. According to Table S5, our methods outperform corresponding baselines on most of evaluation metrics.

**Table S6** Top 15 ADRs ranked by AUC

| ADR concept ID | ADR name | AUC | | AUPR | | Precision | | Recall | | Accuracy | | F1 | |
| --- | --- | --- | --- | --- | --- | --- | --- | --- | --- | --- | --- | --- | --- |
|  |  | MGPS | LP-MGPS | MGPS | LP-MGPS | MGPS | LP-MGPS | MGPS | LP-MGPS | MGPS | LP-MGPS | MGPS | LP-MGPS |
| 35104877 | Febrile neutropenia | 0.928 | 0.933 | 0.534 | 0.537 | 0.519 | 0.519 | 0.692 | 0.692 | 0.955 | 0.955 | 0.593 | 0.593 |
| 35104100 | Aplasia pure red cell | 0.808 | 0.84 | 0.137 | 0.139 | 0.462 | 0.462 | 0.182 | 0.182 | 0.958 | 0.958 | 0.261 | 0.261 |
| 35104694 | Thrombotic thrombocytopenic purpura | 0.846 | 0.834 | 0.186 | 0.187 | 0.3 | 0.3 | 0.353 | 0.375 | 0.969 | 0.97 | 0.324 | 0.333 |
| 37219844 | Pulmonary fibrosis | 0.804 | 0.805 | 0.209 | 0.21 | 0.297 | 0.297 | 0.324 | 0.333 | 0.941 | 0.942 | 0.31 | 0.314 |
| 35204977 | Torsade de pointes | 0.793 | 0.8 | 0.294 | 0.297 | 0.377 | 0.377 | 0.469 | 0.469 | 0.923 | 0.923 | 0.418 | 0.418 |
| 35104302 | Haemolysis | 0.779 | 0.785 | 0.063 | 0.064 | 0.455 | 0.455 | 0.109 | 0.109 | 0.943 | 0.943 | 0.175 | 0.175 |
| 35707713 | Pancreatitis | 0.778 | 0.779 | 0.515 | 0.517 | 0.731 | 0.736 | 0.432 | 0.433 | 0.712 | 0.712 | 0.543 | 0.545 |
| 35909513 | Acute hepatic failure | 0.777 | 0.771 | 0.057 | 0.057 | 0.429 | 0.429 | 0.08 | 0.079 | 0.907 | 0.906 | 0.135 | 0.133 |
| 35909618 | Hepatitis fulminant | 0.755 | 0.769 | 0.063 | 0.064 | 0.294 | 0.294 | 0.114 | 0.114 | 0.938 | 0.938 | 0.164 | 0.164 |
| 36009724 | Stevens-Johnson syndrome | 0.761 | 0.767 | 0.499 | 0.503 | 0.809 | 0.809 | 0.418 | 0.422 | 0.673 | 0.677 | 0.551 | 0.555 |
| 36009754 | Toxic epidermal necrolysis | 0.752 | 0.76 | 0.402 | 0.408 | 0.709 | 0.709 | 0.407 | 0.407 | 0.724 | 0.724 | 0.517 | 0.517 |
| 35607441 | Blindness | 0.759 | 0.759 | 0.13 | 0.13 | 0.395 | 0.395 | 0.185 | 0.185 | 0.893 | 0.893 | 0.252 | 0.252 |
| 35707714 | Pancreatitis acute | 0.759 | 0.759 | 0.214 | 0.214 | 0.146 | 0.146 | 0.667 | 0.667 | 0.954 | 0.954 | 0.24 | 0.24 |
| 35708302 | Intestinal perforation | 0.737 | 0.746 | 0.146 | 0.148 | 0.37 | 0.37 | 0.222 | 0.222 | 0.937 | 0.937 | 0.278 | 0.278 |
| 37019318 | Renal failure | 0.743 | 0.744 | 0.447 | 0.448 | 0.599 | 0.599 | 0.471 | 0.471 | 0.735 | 0.735 | 0.528 | 0.528 |

[1] Atias, Nir, and Roded Sharan. "An algorithmic framework for predicting side effects of drugs." Journal of Computational Biology 18.3 (2011): 207-218.
